# Clinical outcome of Montelukast Sodium in Children with Adenoid Hypertrophy

**DOI:** 10.12669/pjms.37.2.2670

**Published:** 2021

**Authors:** Syed Ali Naqi, Ahmad Hassan Ashfaq, Mumtaz Ahmad Umar, Jais Kumar Karmani, Naveed Arshad

**Affiliations:** 1 Prof. Dr. Syed Ali Naqi, FCPS (ENT). Professor & HOD ENT, Islamabad Medical and Dental College, Islamabad Pakistan; 2 Dr. Ahmad Hassan Ashfaq, FCPS (ENT). Associate Prof., Rawalpindi Medical University, Rawalpindi, Pakistan; 3 Dr. Mumtaz Ahmad Umar, FCPS (ENT). Assistant Professor, Islamabad Medical and Dental College, Islamabad Pakistan; 4 Dr. Jais Kumar Karmani, MD (Medicine). Assistant Professor, Islamabad Medical and Dental College, Islamabad Pakistan; 5 Dr. Naveed Arshad, M.Phil. (Rehabilitation Sciences). Assistant Professor, Islamabad Medical and Dental College, Islamabad Pakistan

**Keywords:** Adenoid, Hypertrophy, Obstructive sleep apnea syndrome, Montelukast sodium

## Abstract

**Background & Objectives:**

Generally, the blockage of upper respiratory tract in children is seen with the hypertrophy of adenoids and tonsils. Normally for patients with adenoid hypertrophy (AH), Adenoidectomy with or without Tonsillectomy is carried out, however it has its own complications like haemorrhage and recurrence of adenoid tissue. Consequently, therapeutic approach has increased extraordinary consideration rather than surgical procedure. The inflammatory process proposed for AH has prompted the utilization of anti-inflammatory drugs to treat this issue. The objective of this study was to assess the impacts of Montelukast sodium in children with enlarged adenoids.

**Methods:**

A randomized controlled trail was performed from April 2018 to March 2019 in the Otorhinolaryngology clinic of Dr. Akbar Niazi Teaching Hospital, Islamabad. In this randomized, placebo treatment-controlled trial, 60 children aged 4-12 years meeting inclusion criteria were isolated into two groups. The study group was treated with Montelukast sodium 5mg consistently for three months while the control group got placebo treatment for a similar timeframe. A questionnaire was filled by parents/ guardians of every child before and after the intervention to evaluate the severity of sleep discomfort, snoring and mouth breathing.

**Results:**

Following 3 months of treatment, significant reduction in size of the adenoids was seen in 76% of study group compared with just 3% of control group getting placebo treatment.

**Conclusion:**

Montelukast sodium seems to be effective in the reduction of the size of adenoids and improvement in clinical manifestations. It can be viewed as a viable option in contrast to surgical treatment in children with hypertrophy of adenoids.

## INTRODUCTION

Adeno-tonsillar hypertrophy is the most widely recognized reason for upper respiratory tract blockage in youngsters with a pervasiveness of 2-3%.^1^ The adenoid is a significant part of Waldeyer’s ring situated in nasopharyngeal region. Due to its extraordinary location, in adjacent choanae and Eustachian tube, it is regularly the site of beginning of numerous medical issues in youth. Adenoid is small in size at early stages, increases in initial four years of life because of the improvement of immunity. Whenever left untreated, AH may prompt obstructive sleep apnea (snuffling, uneasiness, mouth breathing), ear issues, pulmonary hypertension, craniofacial peculiarities and inability to thrive.^2^

However, diagnosis can be thought provoking in a patient with AH symptoms. There are list of recommendations for the diagnosis of AH but recent studies suggested that X-rays soft tissue neck lateral view and nasal endoscopy either rigid or flexible are the gold standard investigations for AH diagnosis.^3^ Enlarged adenoids are removed by adenoidectomy in pediatric age group but still may have post-operative complications like immediate and late hemorrhage (3-5%) and reappearance of adenoid mass (10-20%) of cases.^1^

Significant expenses, absence of child from school, risk of anesthesia and other surgical procedure related morbidities have additionally featured the need of medicinal treatment for adenoid hypertrophy.

Levels of serum immunoglobulins have been seen to be lower in patients who have experienced adeno-tonsillectomy than in healthy control group.^4^ AH may create fundamental irritation in kids and grown-ups as showed by a rise in C-reactive protein and this is connected with psychological and cardiovascular morbidity which diminishes following adeno-tonsillectomy. Leukotrienes are the main factor of inflammatory marker in the respiratory system. These mediators are engaged with the pathogenesis of childhood illness, for example, asthma and adenoid hypertrophy. Human cysteinyl-leukotriene receptor-1 is markedly raised in the tonsillar tissue of youngsters with obstructive sleep apnea (OSA). Likewise, cysteinyl leukotriene receptor-1 which associates with leukotrienes and intervenes the provocative pathway was over communicated in adeno-tonsillar cells and tissues got from youngsters with AH.^5^ Subsequently, leukotrienes anti-inflammatory agents with safe therapeutic profile may provide an effective interventional alternative to adeno tonsillectomy. Montelukast is FDA endorsed oral, cysteinyl-leukotriene receptor which is powerful and very much endured preventive remedy in asthma and allergic rhinitis in children over one year. Additionally, Montelukast has not prompted resistance in long term studies.^6^,^7^

The rationale behind this study was to evaluate the theory that Montelukast treatment may prompt improved nighttime symptoms, quality of life, and anatomical characteristics just as endoscopic and radiologic outcomes in youngsters with adenoid hypertrophy.

## METHODS

This randomized controlled trial was performed from April 2018 to March 2019 in the Otorhinolaryngology clinic of Dr. Akbar Niazi Teaching Hospital, Islamabad. The approval was taken from Ethical Committee of the Hospital vide letter number of IRB. No. 1.60.IMDC-2017. Sixty consecutive children meeting the inclusion criteria were enrolled. Analysis was based on clinical assessment, lateral neck radiograph, Flexible nasal endoscopy and X-rays post nasal space (soft tissue neck lateral view). Children more than four and less than 12 years old with snoring, grade 3 or more noteworthy nasopharynx deterrent on endoscopic assessment and 50% or more in Adenoidal/Nasopharyngeal proportion in radiographic examinations were included in this study. Obesity characterized as BMI for age percentile ≥ 1.645 (95%), genetic irregularities, previous or current utilization of montelukast, acute infection of upper respiratory tract, any anti-microbials or corticosteroids use a month prior and children with previous history of adeno-tonsillectomy were excluded from the study.

Children were examined by a particular otorhinolaryngologist and were allotted to study or control group (n=30 each). The study group got Montelukast four and 5mg once a day for children less than 6 and more than 6 years old respectively. While placebo treatment tablets were given for the control group. All guardians were instructed to give the tablets at sleep time. Second nasal endoscopy alongside X-rays neck was performed following three months of treatment.

To evaluate the patency of airway, lateral neck radiographs were performed utilizing the standard system in the radiology department at Dr. Akbar Niazi Teaching Hospital Islamabad. X-ray lateral neck was performed before the start of study and repeated following three months treatment course. Flexible nasal endoscopy was performed to acquire a full choanal picture. Topical anesthesia and vasoconstrictors were introduced in all patients undergoing nasal endoscopy. The measurement of obstruction was classified utilizing Parikh technique, which depends on anatomical relationship. Evaluation 0 = None, Evaluation 1 = Torus tubarius, Evaluation 2 = Torus tubarius and vomer, Evaluation 3 = Vomer and soft palate.

Flexible nasal endoscopy was performed at the beginning and end of study. The primary outcome was mouth breathing and restlessness. Secondary result measures were the adenoid size based on naso-endoscopy and lateral neck soft tissue radiograph. Based on the severity of symptom, a score (0-3) was recorded; 0-Never existed, 1-Intermittent manifestations, 2-Present occasionally, 3-Constantly present.All numerical data was subjected to statistical analysis with Mann Whitney U Test. P ≤ 0.05 was considered significant.

## RESULTS

Sixty children meeting the inclusion criteria were included. No withdrawals or any reactions were seen in any of the patients. Out of 30 children in study group (Montelukast), 19 (63.33%) were males and 11 (36.67%) were females with mean age 6.9±2.33 years. Whereas, in placebo group, out of 30 children 15 (50%) were males and 15 (50%) were females with mean age 6.83±2.36 years.

**Fig.1:**
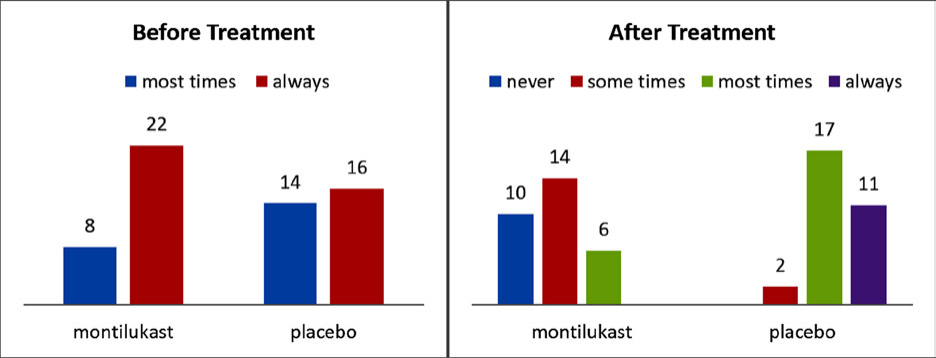
Snoring scores before and after treatment.

The primary symptoms were open mouth breathing, snoring and restlessness. As indicated by the Mann Whitney U Test, no distinction was seen in snoring between the two groups (p=0.111). Three are the mean score in each group. However, a significant difference was seen between the two groups after treatment (p ≤ 0.007) (Fig.1).

**Fig.2:**
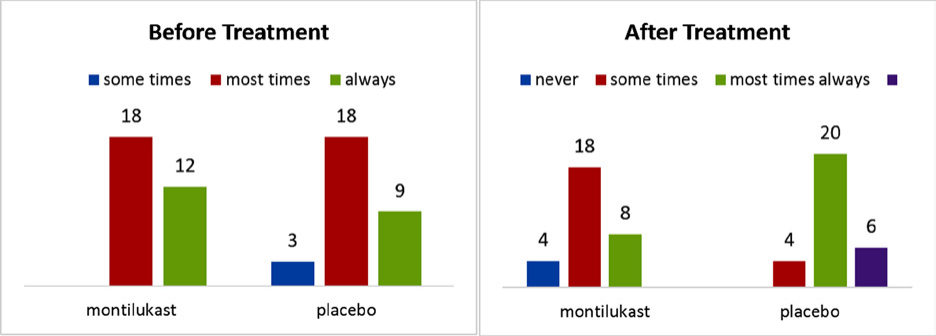
Before and after treatment of sleep discomfort score.

Regarding restlessness, no significant difference between the two groups was seen toward the start of study (p=0.408). However statistically significant difference observed after treatment (p ≤ 0.0001) (Fig.2).

**Fig.3:**
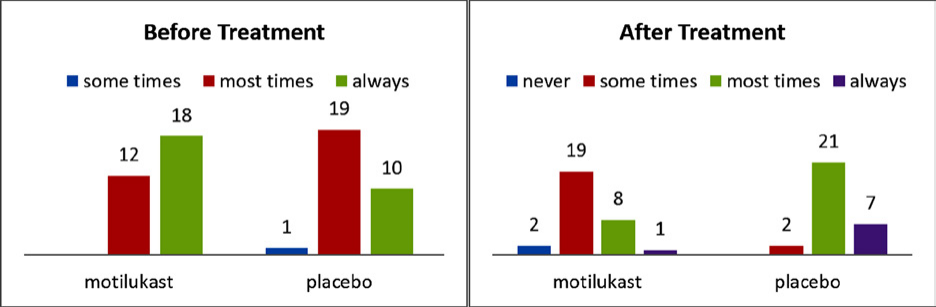
Before and after treatment of mouth breathing score.

The outcomes were comparable for mouth breathing, demonstrating a statistically significant difference after the treatment period (p=0.33 versus p ≤ 0.0001). For the indication of mouth breathing, the mean score of the treatment group was higher toward the beginning of study, yet the little distinction was not measurably significant (p=0.33) (Fig.3).

Nasal endoscopy and lateral neck radiography were additionally used to ponder the patient’s effects toward the beginning and end of treatment course. The mean score between the two groups showed an important distinction before the treatment (p=0.03), yet a progressively significant difference was seen after the treatment course (p ≤ 0.0001) on nasal endoscopy.

In radiographic examinations, a marked decrease of ≥25% was seen in the size of adenoids after treatment. The response rate was 76% in the treated group and 3.3% in placebo group. P ≤ 0.0001 was considered measurably significant.

Correlation of these two clinical tests in connection to the clinical effects was furnished utilizing Cohen’s Kappa coefficient which is used to measure inter rater reliability. This uncovered an estimation of 0.55 for radiography and 0.8 for naso-endoscopy. Subsequently, naso-endoscopy had a solid relationship with the patient’s manifestations and his/her general condition (Table-I).

**Table-I:** Correlation of clinical tests (Endoscopy vs Radiography) between two groups.

	*Montelukast*		*Placebo*	

	*Before*	*Afte*	*Before*	*After*
Endoscopy (score)	3.77±0.43	2.37±0.77	3.57±50	3.43±0.57
Radiography (%)	87.23±8.97	51.33±5.91	81.16±7.27	77.83±9.06

* *P ≤ 0.0001*

## DISCUSSION

Adenoids are lympho-epithelial organs confined to the top of nasopharynx region. It typically reaches at their most extreme size between three to six years and then regresses. Adenoid hypertrophy frequently follows upper respiratory tract infection and is a typical illness of childhood. Chronic infections are the most likely recognized signs of physiological and pathological changes in the adenoids. It raises nasal blockage, mouth breathing, restlessness, contaminations in the ear and sinuses. Normally adenoid tissue relapses with the progression of time if the infection doesn’t occur.^8^ Adenoid tissue may regrow even after adenoidectomy because of chronic hypersensitive responses or infections. Because of surgical procedure related intricacies like massive bleeding, velopharyngeal insufficiency and risks of anesthesia, alternative treatment methodologies have grown over time.

Our study exhibited that Montelukast sodium in the form of oral, chewable tablet given to the children with AH for three months has successfully diminished the seriousness of breathing difficulty, snoring and mouth breathing along with decrease in the size of adenoid tissue. Moreover, this treatment endured by the patients with no side effects. Notable decrease in adenoid size was affirmed through lateral neck soft tissue X-rays and naso-endoscopy. In X-rays adenoidal/nasopharyngeal proportion was estimated utilizing the technique depicted by Fujioka and colleagues.^9^,^10^ Despite certain difficulties and preforming endoscopy, it has a stronger correlation with clinical symptoms.^11^ Shokouhi et al.^12^ also observed a significant reduction in the size of adenoids in a group receiving Montelukast which supports our study.

In a study by Goldbart et al,^13^ Montelukast sodium was utilized in the treatment of obstructive sleep apnea in forty children between four to 12 years. 20 children received 4mg chewable tablets of Montelukast sodium for children less than six years old and 5mg for those greater than six years while the other twenty got placebo treatment both for three months.

A noteworthy improvement of more than 50% was seen in polysomnographic parameters and the Adenoidal / Nasopharyngeal proportion in radiography decreased from 81% to 57% (Cohen kappa coefficient 0.55). The difference in the outcomes may be because of varied measurements of medication utilized 4mg for children less than 6 years and 5 mg for more than six years of age in our study or the distinction in the severity of manifestations towards the start of study. We found moderate to severe symptoms in our study while in the previously mentioned study; the manifestations were mild to moderate. Tuhanioglu B and Erkan SO^14^ additionally discovered relapse in the adenoid size in the group who took Montelukast sodium. The clinical significance of our study was the symptoms of adenoid hypertrophy assessed showed improvement in sleep and may have a major impact on quality of life. The results of this study support the utilization of a leukotriene modifier as a safe therapeutic alternative for the treatment of children with AH and thus can be recommended as a substitute to surgical procedure to prevent post-operative complications.

### Recommendations of the study

Before this approach can be accepted as a medical standard of care, large-scale studies are warranted to further reinforce our findings and new developments of FDA warnings and neuropsychiatric adverse effects; caution needs to be exercised to reserve its use for the selected patients.

### Limitations of the study

Difficult to attained the proper view of X-rays in small children and difficulty in performing endoscopy in some children.

## CONCLUSION

A notable decrease was observed in the size of adenoid tissue along with improvement in the clinical symptoms with Montelukast sodium treatment. So it is considered as an effective substitute to surgical procedure in children with enlarged adenoids.

### Authors’ Contribution:

**SAN** provided concept/research design and did data collection, subjects & editing of manuscript.

**MAU, JKK and NA** did statistical analysis and manuscript writing.

**JKK and AHA** did editing of manuscript and project management.

**SAN and MAU** did data collection, subjects and provision of facilities/equipment.

**SAN and NA** take the responsibility and is accountable for all aspects of the work in ensuring that questions related to the accuracy or integrity of any part of the work are appropriately investigated and resolved.
